# Prevalence of Musculoskeletal Pain and Its Relation With Weight of Backpacks in School-Going Children in Eastern India

**DOI:** 10.3389/fpain.2021.684133

**Published:** 2021-08-18

**Authors:** Sindhu Sankaran, Joseph John, Sameer Sekhar Patra, Rashmi Ranjan Das, Amit Kumar Satapathy

**Affiliations:** Department of Pediatrics, All India Institute of Medical Sciences Bhubaneswar, Bhubaneswar, India

**Keywords:** backpack, back pain, musculoskeletal pain, bag weight, school bag

## Abstract

**Background:** Recently, heavy school backpacks have become a significant concern among parents and health professionals, as well as the media, but evidence for the same is limited in the Indian context.

**Aim:** To find the prevalence of musculoskeletal pain among school-going children and its relationship with backpack weight.

**Design:** Cross-sectional study.

**Method:** This study was carried out among school-going children from grade 6 to 10 with age of 10 to 16 years from an urban and rural location. Schools were selected randomly from all enlisted schools in the district of Khurdha, Odisha state of India. A structured questionnaire was administered to assess symptoms of musculoskeletal pain. Anthropometric measurements along with backpack weight were taken.

**Statistical Analysis:** Chi-square test was performed for categorical variables and Student's *t*-test for continuous variables. Multivariate regression analysis was performed to identify factors with maximum effect on musculoskeletal pain.

**Results:** The prevalence of musculoskeletal pain was 18.8% in the preceding year. Backpacks weights were higher among children of urban schools as compared with rural areas. Children from urban schools were more likely to have pain than those from rural schools (OR 1.88, 95% CI 1.41–2.49). Those children with a backpack weight more than 10% of body weight had almost twice the risk of musculoskeletal pain compared to backpack weight less than 10% (OR 1.91, 95% CI 1.4–2.6) in univariate analysis where as no significant association was found on multivariate analysis.

**Conclusion:** The prevalence of musculoskeletal pain was high in school-going children. In children, carrying higher backpack weight, and a higher percentage of the backpack to bodyweight had a significant association with musculoskeletal pain. Gender, height, body mass index, and backpack weight to body weight > 10% had no association with musculoskeletal pain.

## Introduction

The age of school-going children ranges between 6–16 years, and these children go through various physical and psychosocial developmental stages and phases of accelerated growth of skeletal and soft tissue, especially during puberty. Any repetitive stress in this age group may have far-reaching consequences into adulthood. Recently, heavy school backpacks have become a great concern among parents, health professionals, and the media (1, 2). Some studies suggest that with the use of heavy school backpacks, there is an increase in the incidence of low back pain, abnormal posture, and other musculoskeletal problems (3–8). Children with Backpack weight > 10% of their body weight has higher prevalence musculoskeletal pain than those with backpack weight < 10% (9). On the contrary, some other studies suggest that although there is a high prevalence of back pain among children, it has no association with the weight of backpacks carried and may be due to other factors than mechanical ones (10–13).

Many countries, such as India, have tried to ascertain a specific percentage of body weight to restrict the load carried by children in their school backpacks (14, 15). The European countries have backpack limits of 10% of body weight, while American occupation therapy recommends a limit of 15% body weight, but these recommendations are also highly diverse and there are no set international guidelines on this subject. Some studies have also claimed that there is a gender variation and that the body mass index (BMI) plays an essential role in the causation of low back pain among school-going children, although there is not enough concrete evidence to justify this (5, 6). In India, the Ministry of Human Resource Development has recommended a bag weight of up to 5 kg for children in grade 10, 4.5 kg for grades 8 and 9, and 4 kg for grades 6 and 7. Previous studies have reported having a higher backpack for private schools as compared to public schools. The impact of the backpack on musculoskeletal pain (MSP) with respect to public and private schools has not been well-established though. Thus, with such varied literature, it is perplexing to healthcare professionals and others alike if there is a need for putting limitations of weights that children can carry in their backpacks and what amendments should be included in the limitations that should be put depending on the demographic profile of these children. There is limited literature on the burden of MSP and its associated factors in India, specifically in rural areas from the eastern part of India. This study aimed to determine the prevalence of musculoskeletal pain in school-going children and its relation to backpack weight.

## Methods

A cross sectional study on school-going children from urban and rural locations was conducted. Participating schools were selected randomly from the enlisted schools in the district of Khurdha, Odisha state of India. Every school within the Bhubaneswar Municipal Corporation limits was classified as urban and the others as rural. A number was assigned to all schools, and selection was done randomly. After ethical clearance by the institute ethics committee and permission from the school authorities and parents was obtained, students from the 6 to 10 grade willing to participate in the study were included. Children were excluded if they had any of the following: (i) any evidence of congenital or inherited musculoskeletal disorders (ii) using other forms of school bags other than backpacks like a bag with wheels etc. After explaining the details of the study, a structured questionnaire was administered, and anthropometric measurements were taken. The questionnaire was explained to the students, and their understanding was checked by asking them their understanding of each question. Investigators helped the students by simplifying the questions and clarifying doubts that arose.

### Sample Size

A previous Indian study found the prevalence of low back pain in the pediatric population to be 53.9% (12). Keeping this in mind, we calculated the sample size by the formula Z^2^*p^*^(1 – p)/I^2^; where Z is the 95% confidence interval, p is the prevalence taken (53.9%), and I is the relative error (taken as 10% in this study) that is 5.39. So, the sample size calculated was 329. Because of the cluster sampling technique, a design factor of 4 was multiplied to reach the final sample size of 1,316.

### Data Collection

Demographic details, such as name, date of birth (DOB), gender, and grade and section were collected. Anthropometric measurements including height, weight of child, and weight of child with backpack and other accessories were obtained. A digital electronic scale with an accuracy of 0.01 kg calibrated over a range of known weights, and a stadiometer accurate to 0.1 cm were used for anthropometric measurements. The weight of each student was measured first without schoolbag, then after, carrying his schoolbag, which also included a tiffin box and water bottle inside it, to obtain the total weight. The difference of the two weights was recorded as the schoolbag weight, and then, the schoolbag weight percentage compared with the body weight was calculated. Similarly, height was determined using the stadiometer with students standing straight on it without shoes and looking at Frankfurt plane with heel, buttock, shoulder and occiput touching the stadiometer. BMI was calculated using formula weight (kg)/(height in mt)^2^.

To assess for musculoskeletal symptoms, the Modified Nordic Musculoskeletal Disorders Questionnaire was used (16). This validated questionnaire has a clear image showing different body areas labeled for easy understanding especially for children.

Data were analyzed using the STATA software, Chi-square test for categorical variables, and student's *t*-test for continuous variables. Multivariate regression analysis was performed to identify factors having a maximum effect on musculoskeletal pain. A *p*-value of <0.05 was considered significant.

## Result

We collected data of 1,329 children across four different (two urban and two rural) categories of schools from 10 to16 years and between grades 6 and 10. Out of these 1,329 children, 685 children belong to an urban area, whereas 644 children were from a rural area. We collected data from both public (680 children) and private (649 children) schools. Thus, a total of four categories of children were accounted for—urban public, urban private, rural public, and rural private schools. The number of children belonging to urban private, urban public, rural private, and public school are mentioned in above (Table 1).

**Table 1:** Demographic details of school children.

**Demographic details**	**Urban (mean ± SD)**	**Rural (mean ± SD)**	***P*-value**
Number of children	685	644	
Age	14.37 ± 1.16	13.17 ± 1.34	
**Sex**			
Boy	353	310	
Girls	332	334	
**Type of school**			
Public	348 (26%)	332 (25%)	
Private	337 (25%)	312 (24%)	
Weight (kg)	52.5 ± 13.6	44.8 ± 11.7	<0.05
Height (mt)	1.57 ± 0.09	1.52 ± 0.09	
BMI (kg/m^2^)	21.01 ± 4.43	19.26 ± 4.90	
Weight of backpack (kg)	4.62 ± 1.73	3.49 ± 1.26	<0.05
Percent backpack to body wt (%)	7.55 ±3.06	8.27 ±2.73	<0.05

The mean age of urban school children was 14.4 years whereas that of rural school was 13.4 years. The mean weight of children of urban schools was 52.5 kg (±13.6) kg, and that of rural schools was 44.8 kg (±11.7).

We measured the backpack weight of both urban and rural children and looked for the association of backpack weight with musculoskeletal manifestation in the form of pain in shoulder joint and elbow joint, neck and back. The mean weight of backpacks of the children in the urban schools was 4.62 (±1.74) kg, whereas that in the rural school was 3.5 (±1.26) kg (Table 2). The mean backpack weight difference among children from private (4.76 ± 1.63 kg) and public (3.42 ± 1.33 kg) schools was statistically significant. When backpack weight was calculated in terms of percentage of body weight, the urban school children (7.55 % ± 3.06) had a lower percentage as compared with the rural school children (8.27% ± 2.73) (Table 1). This probably was secondary to higher body weight in urban school children as compared with rural schools. When compared among various grades in private and public schools, the difference was significant except for the 6 grade (Table 3).

**Table 2:** Association of backpack weight among urban vs. rural, public vs. private schools and various grades.

**Characteristics**	**Weight of backpack (kg) (mean ± SD)**	***P*-value**
**Residence**		
UrbanRural	4.62 ± 1.74 3.5 ± 1.26	<0.05
**Type of school**		
PublicPrivate	3.42 ± 1.33 4.76 ± 1.63	<0.05
**Grade**		
6th	4.08 ± 1.309	
7th	4.43 ± 1.64	
8th	4.05 ± 1.76	0.012
9th	4.09 ± 1.47	
10^th^	3.86 ± 1.77	

**Table 3:** Association of backpack weight among public vs. private schools across grades.

**School grades**	**Weight of backpacks (kg)**	**Weight of backpacks(kg)**	***P*-value**
	**Public school**	**Private school**	
	**(Mean ± SD)**	**(Mean ± SD)**	
6th	3.91 ± 1.38	4.13 ± 1.29	0.40
7th	3.72 ± 1.41	5.05 ± 1.56	<0.05
8th	3.26 ± 1.42	5.06 ± 1.63	<0.05
9th	3.56 ± 1.09	5.06 ± 1.63	<0.05
10th	3.23 ± 1.36	5.16 ± 1.89	<0.05

The frequency of pain in different groups of children in urban and rural areas is given in Table 4. There were 250 (18.8%) children who experienced any degree of pain the preceding year. The major sites of pain reported in this study were the shoulder joints (39.2%), knee (19.6%), back (18%), and neck (10.4%). Children from urban and private schools had a higher prevalence of pain (Table 4); 175 out of 1,056 children (16.6%) with backpack weight < 10% of body weight has musculoskeletal pain whereas 75 out of 273 children (27.5%) with backpack weight more than 10% of bodyweight has musculoskeletal pain. Mean backpack weight, percentage of backpack weight to body weight, backpack weight more than recommended weight, and backpack weight more than 10% of body weight are significantly associated with musculoskeletal pain in univariate analysis (Table 4).

**Table 4:** Risk factors associated with musculoskeletal pain.

**Parameter**	**Musculoskeletal pain—present *N* (%)**	**Musculoskeletal pain—absent *N* (%)**	**Odds ratio (OR) 95% CI**	**Adjusted odds ratio 95% CI**	***P*-value**
**Resident**					
UrbanRural	160 (64%) 90 (36%)	525 (48.7)554 (51.3)	1.88 (1.41-2.49)	0.93 (0.42-2.03)	<0.001
**School**					
PublicPrivate	117 (46.8%) 133 (53.2%)	563(52)516 (48)	0.81 (0.61-1.06)	0.82 (0.58-1.05)	0.12
**School type**					
Urban publicUrban privateRural publicRural private	64 (25.6%) 96 (38.4%) 53 (21.2%) 37 (14.8%)	284 (26.3)241 (22.3)279 (25.9)275 (25.5)	N/A	N/A	N/A
**Sex**					
GirlsBoys	136 (54.4%) 114 (45.6%)	530 (51)549 (49)	1.24 (0.94-1.63)		0.13
**School grade**					
6th7th8th9th10th	13 (5.20%) 28 (11.20%) 45 (18.00%) 117 (46.80%) 47 (18.80%)	118 (10.9)142 (13.1)287 (26.6)306 (28.4)226 (21.0)	N/A	N/A	N/A
**Obese vs. Non-obese**					
Non-obese Obese	195 (78) 55 (22)	874 (81)205 (19)	0.83 (0.59-1.16)	1.00 (0.67-1.50)	0.28
**Recommended wt for**					
**grades (HRD Ministry)**					
BelowAbove	124 (49.6) 126 (50.4)	728 (67.5)351 (32.5)	0.47 (0.36-0.63)	0.78 (0.61-1.55)	<0.001
**Backpack % to body weight**					
<10%>10%	175 (30) 75 (70)	881 (18)198 (82)	1.91 (1.4-2.6)	0.95 (0.62-1.45)	<0.001
**BMI**					
OverallUrbanRural	20.53 ± 4.44 21.59 ± 4.32 18.85 ± 4.04	20.07 ± 4.820.83 ± 4.419.35 ± 5.02	N/A	N/A	0.16 0.05 0.21
Mean weight of backpack (kg)	4.74 ± 1.83	3.92 ± 1.58	N/A	N/A	<0.05
Mean backpack % to body weight	8.67 ± 3.23	7.72 ± 2.8	N/A	N/A	<0.05

*Risk factor associated with pain after univariate (OR) and multivariate analysis (Adjusted OR). OR, Odds ratio; CI, Confidence interval; N/A, Not applicable*.

A multivariate regression analysis was performed to assess the relationship between back pain and various independent variables such as gender, school type (private vs. public school), backpack weight, percentage backpack, obesity, and back pack weight more than recommended weight. In logistic regression, a significant association was found between the weight of backpack and musculoskeletal pain in children. Gender had no association with pain. Children having backpack weight higher than that recommended (as per HRD ministry), or with backpack weight more than the recommended weight or higher percentage of backpack to body weight did not have any association with pain in multivariate analysis (Table 4).

## Discussion

There is a growing concern among parents regarding the increase in school bag weight in children of school-going age. There is very limited evidence on excessive backpack weight causing health issues in children. There are few studies published that have addressed this growing concern. It has been found in studies that backpack weight significantly contributes to musculoskeletal pain in the form of back pain, shoulder pain, and neck pain. The data from India are further limited. Although the Ministry of Human Resources and Development (HRD) has recent recommendations for backpack weight for various grades, its effect on the musculoskeletal system is not well-studied.

In this study, we found the prevalence of musculoskeletal pain among school children to be 18.8%, whereas the prevalence was reported to be 35.4% in a study from Uganda (3) while that was documented to be 40% in school children in a meta-analysis done by Calvo-Munoz et al. (17). The backpack weight is significantly high in children from private schools and among urban schools. This result is similar to that of a study from Uganda conducted by Mwaka et al. (3). This may be due to students of urban schools carrying more textbooks along with tiffin and lunch box and water bottles as compared with their rural counterparts in public schools. As per this study, shoulder, knee, and neck are the commonly reported sites of pain by children, which is similar to a study published by Parthibane et al. (18). We did not find any significant gender difference in the prevalence of musculoskeletal pain in school children as compared with previous studies where boys were reported to have a lower risk of pain than girls (19, 20). Boys perhaps reported a higher prevalence of pain in view of greater exposure to sports and intense activities, whereas Akbar et al. had reported a higher prevalence in young females (21). This may be due to a higher perception of pain as reported by Kovacs et al. (22). We did not find BMI to be a risk factor for developing pain in school children, although previous studies have reported an association (7). Children using a heavier backpack were at a higher risk of having musculoskeletal pain. This was unlike that reported previously, where no association was found between backpack weight and pain (23). Similarly, we did not find any association between the percentage of backpack weight to body weight with musculoskeletal pain. Although many professional occupational bodies worldwide, along with the American Academy of Pediatrics, recommend backpack weight not to exceed 11-15% of body weight, we did not get any significant association in this study. Similarly, we also could not find any association between musculoskeletal pain and the maximum recommended backpack weight by the HRD ministry. Cohort studies will be better to investigate such association of backpack weight and musculoskeletal pain.

### Limitation

We collected data from the students themselves; parents were not involved in data collection although consent was taken *a priori*. We did not conduct a detailed clinical examination, nor did we take a detailed history of injury, lifestyle, sitting posture, etc. as other factors contributing to the pain.

## Conclusion

The prevalence of musculoskeletal pain among school children was found to be 18.8%. Children carrying a heavier backpack and with a higher percentage of backpack to body weight had a significant association with MSP. Gender and backpack weight to body weight > 10% had no association with musculoskeletal pain. The current HRD ministry-recommended backpack weight for each grade was noted to have a higher risk of having pain as per our study. Hence, more studies are needed to determine the appropriate maximum backpack weight for school children of different grades.

## Data Availability Statement

The raw data supporting the conclusions of this article will be made available by the authors, without undue reservation.

## Ethics Statement

The studies involving human participants were reviewed and approved by Institute Ethics Committee, AIIMS Bhubaneswar. Written informed consent to participate in this study was provided by the participants' legal guardian/next of kin.

## Author Contributions

SS and SP were involved in data collection and entry, participated in data analysis, and wrote the first draft of the manuscript. JJ conceptualized the study, coordinated the execution of the project, critically reviewed the draft manuscript, and acted as guarantor. RD and AS supervised data collection, interpreted the data, and critically reviewed the draft manuscript. All the authors have approved the final version of the manuscript.

## Conflict of Interest

The authors declare that the research was conducted in the absence of any commercial or financial relationships that could be construed as a potential conflict of interest.

## Publisher's Note

All claims expressed in this article are solely those of the authors and do not necessarily represent those of their affiliated organizations, or those of the publisher, the editors and the reviewers. Any product that may be evaluated in this article, or claim that may be made by its manufacturer, is not guaranteed or endorsed by the publisher.
