# Supplementary material for: Prevalence of Musculoskeletal Pain and Its Relation With Weight of Backpacks in School-Going Children in Eastern India
Source: Front Pain Res (Lausanne). 2021 Aug 18;2:684133. doi: 10.3389/fpain.2021.684133 (PMC8915625; doi:10.3389/fpain.2021.684133)
Supplement: Supplementary file 1 [file Data_Sheet_1.PDF]

How to answer the questionnaire:

Picture: In this picture you can see the approximate position of the parts of the body referred to in the table. Limits are not sharply defined, and certain parts overlap. You should decide for yourself in which part you have or have had your trouble (if any).

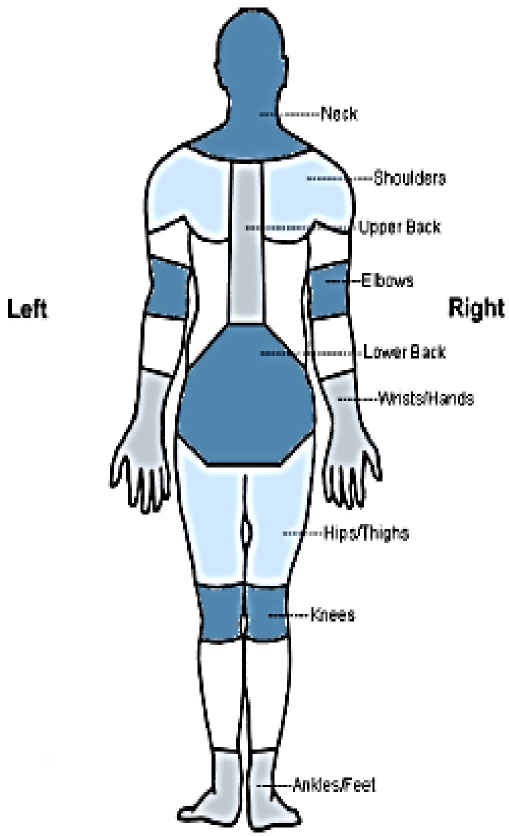

Back View

Table: Please answer by putting an "X" in the appropriate box - one "X" for each question. You may be in doubt as to how to answer, but please do your best anyway. Note that column 1 of the questionnaire is to be answered even if you have never had trouble in any part of your body; columns 2 and 3 are to be answered if you answered yes in column 1.

**To be answered by everyone**	**To be answered by those who have had trouble**	
Have you at any time during the last 12 months had trouble (ache, pain, discomfort, numbness) in:	Have you at any time during the last 12 months been prevented from doing your normal work (at home or away from home) because of the trouble?	Have you had trouble at any time during the last 7 days?
**Neck**<br>☐ No ☐ Yes	☐ No ☐ Yes	☐ No ☐ Yes
**Shoulders**<br>☐ No ☐ Yes, right shoulder<br>☐ Yes, left shoulder<br>☐ Yes, both shoulders	☐ No ☐ Yes	☐ No ☐ Yes
**Elbows**<br>☐ No ☐ Yes, right elbow<br>☐ Yes, left elbow<br>☐ Yes, both elbows	☐ No ☐ Yes	☐ No ☐ Yes
**Wrists/Hands**<br>☐ No ☐ Yes, right wrist/hand<br>☐ Yes, left wrist/hand<br>☐ Yes, both wrists/hands	☐ No ☐ Yes	☐ No ☐ Yes
**Upper Back**<br>☐ No ☐ Yes	☐ No ☐ Yes	☐ No ☐ Yes
**Lower Back (small of back)**<br>☐ No ☐ Yes	☐ No ☐ Yes	☐ No ☐ Yes
**One or Both Hips/Thighs**<br>☐ No ☐ Yes	☐ No ☐ Yes	☐ No ☐ Yes
**One or Both Knees**<br>☐ No ☐ Yes	☐ No ☐ Yes	☐ No ☐ Yes
**One or Both Ankles/Feet**<br>☐ No ☐ Yes	☐ No ☐ Yes	☐ No ☐ Yes
